# Study protocol paper for the multi-site feasibility evaluation of mobile and technology-assisted aftercare services for crisis stabilization units

**DOI:** 10.1186/s40814-023-01361-8

**Published:** 2023-07-31

**Authors:** Carrie Pettus, Rachel Fulmer, Shelby D. Pederson, Jacob Eikenberry

**Affiliations:** 1 Wellbeing and Equity Innovations, PO Box 14641, Tallahassee, FL 32317 USA; 2 Institute for Justice Research and Development, Florida State University, 2010 Levy Ave, Suite 3400, Tallahassee, FL 32310 USA; 3 Colorado Mesa University, 1100 North Avenue, Grand Junction, CO 81501-3122 USA

**Keywords:** Mental health disorder, Crisis stabilization, Law enforcement, Substance use disorders, Randomized, Feasibility, Acceptability

## Abstract

**Background:**

Law enforcement frequently responds to substance abuse and mental health crises. Crisis stabilization units (CSUs) operate as a public-receiving facility to provide short-term stabilization services for individuals experiencing these crises and offer law enforcement an important alternative to arrest. However, there is limited understanding about how and when law enforcement decides to use CSUs. There is also the challenge of retaining individuals in treatment after CSU stabilization to prevent future crises and persistent engagement with police. This study will respond to these gaps by exploring CSU procedures and examining the feasibility and acceptability of a technology-assisted mobile aftercare intervention designed for individuals brought to a CSU by law enforcement.

**Methods:**

This study will consist of three aims. Aim 1 will include qualitative interviews with law enforcement and CSU-affiliated mental health staff (*n*=80) regarding CSU utilization and collaboration logistics between the groups. Findings from Aim 1 will be synthesized for the development of an implementation guide of our intervention, mobile, and technology-assisted aftercare, designed for individuals brought to a CSU by law enforcement, during Aim 2. During Aim 2, intervention services will be pilot-tested for 6 months through a small sample (*n*=24), randomized control trial (RCT). Control participants will receive standard services available for individuals discharging from a CSU. Treatment participants will receive the mobile aftercare intervention. Qualitative and quantitative data will be collected at 2 weeks, 3 months, and 6 months post-recruitment for all study participants. Aims 1 and 2 will inform the design of a multi-site RCT to compare CSUs with and without mobile and technology-assisted aftercare (Aim 3).

**Discussion:**

The study will offer decision making and procedural insight into law enforcement use of CSUs as an alternative to jail and provide opportunities to inform that process. This research will provide outcome trends for those who go through standard CSU services compared to those who receive mobile and technology-assisted aftercare services. The current study will inform a larger RCT efficacy study of CSUs with and without technology-assisted aftercare services.

**Trial registration:**

This study was registered on ClinicalTrials.gov (reference #NCT04899934) on May 25, 2021.

## Introduction

Individuals with behavioral health disorders are disproportionally represented in jails and prisons [1–4]. Rates of substance use disorders are approximately nine times higher, and rates of mental health disorders are three to six times higher among incarcerated populations compared to members of the general public [5]. According to the Bureau of Justice Statistics (BJS), 44% of incarcerated individuals have had at least one mental health diagnosis, and 45 to 53% meet substance use disorder criteria [6]. Incarcerating individuals experiencing behavioral health crises exacerbates symptoms and can lead to adverse consequences, such as loss of employment opportunities, loss of housing, and disruption in social connections [7]. Individuals who have mental health or substance use disorders tend to stay in jails longer, have an increased risk for self-harm, and receive more punitive disciplinary actions [8]. Despite the high rates of substance use and mental health disorders among incarcerated populations, individuals often do not receive the needed services in jail due to funding and limitations with staffing [8, 9]. Additionally, jails are not designed to connect people to treatment in the community. After release, these same individuals are likely to continue to have untreated symptoms and have repeated contact with law enforcement officers (LEOs). Research has indicated that LEOs often have reoccurring contact with individuals with mental health concerns [10].

Crisis stabilization units (CSUs) provide short-term, intensive residential support, education, and treatment to manage symptoms of mental health and substance use disorders, otherwise known as behavioral health disorders. The goal of the CSU is to stabilize the individual quickly—often within 72 h—and refer them to community-based systems of care after discharge*.* CSUs are public receiving facilities, meaning when law enforcement brings a patient in under an emergency psychiatric hold; the CSU cannot refuse to accept the patient. Thus, CSUs provide a critical intervention opportunity for individuals in crisis to avoid jail and get the services they need.

CSUs act as a vital pre-booking jail diversion option for law enforcement who frequently respond to mental health and substance-related crises [10]. Pre-booking diversion programs are designed to target an individual’s initial contact with law enforcement and must include the ability to connect that individual to a mental health professional or a 24-h non-refusal crisis center, such as a CSU [1]. Pre-booking jail diversion programs were greatly increased following 1997 due to funding and research initiatives by the Substance Abuse and Mental Health Services Administration (SAMSHA) [1]. However, there was a heavy reliance on hospitals to provide crisis stabilization services rather than CSUs. It was not until the 2010 Affordable Care Act that the development of CSUs was notably incentivized due to the policy encouraging the rerouting of mental health crisis patients away from hospitals and emergency departments [11]. To date, the majority of research conducted has examined the use of CSUs as an alternative to the previous reliance on the medical system and psychiatric hospitalization. Less is known about the use of CSUs by law enforcement as a diversion from jail [11–13].

### CSU aftercare services

While CSUs provide an opportunity to divert individuals in crisis away from jail, they focus on short-term stabilization, and it can be challenging to connect and engage individuals with treatment after CSU discharge. The retention for mental health and substance use treatment services is a common difficulty across the general population but perhaps more pronounced with the typical CSU or outpatient population [14–17]. Olfson et al. [16] found about 1 in five adults in outpatient mental health treatment drop out before completing the recommended amount of treatment. Additionally, a meta-analysis assessing dropout rates of in-person psychosocial substance use disorder treatments found on average 30.4% of individuals drop out of treatment [17]. Dropout rates varied depending on the treatment population, the substance being targeted, and the characteristics of the treatment program. Programs that had longer session lengths and more sessions had higher rates of dropouts. Dropout rates were highest for studies focused on the treatment of cocaine and methamphetamines and lowest for studies focused on the treatment of alcohol, tobacco, and heroin [17]. Addressing barriers to the long-term stabilization of CSU patients is vital to breaking the pattern of repeated exposure to the criminal justice system. A recent analysis of the jail population in Arkansas showed over 33% had utilized a CSU within a year of their admission into jail, suggesting the vital need of continued retention in treatment after a CSU discharge [10, 14]. Significant barriers to the long-term stabilization and mental health treatment often include accessibility and availability [15]. Structural and logistical factors such as transportation, stable housing, costs, and long waitlists for community-based treatment decrease access and use. Attitudinal barriers, such as not perceiving a need for treatment, have also impacted initiating and continuing treatment for individuals experiencing mild to moderate mental health crises [15].

Technology and mobile applications may be a viable solution to addressing these barriers by providing unlimited and remote access to assistance. However, there is limited literature on mobile applications with justice-involved populations diagnosed with a substance use, mental health, or co-occurring disorder [18, 19]. A study using mobile applications to deliver treatment to homeless individuals recently released from jail is expected to complete data collection in May 2022 [20]. A pilot study of mobile app use for drug court participants showed that participants used the recovery support app regularly over 4 months [21]. While there is limited literature and empirical evidence regarding the use of mobile applications with justice-involved individuals, mobile applications have shown promise for effectiveness with individuals with serious mental illnesses [22, 23]. Additionally, a meta-analysis of 66 randomized controlled trials showed mobile applications improved depression (*g*=.028, *n*=54) and anxiety symptoms (*g*=0.30, *n*=39), stress (*g*=0.35, *n*=27), quality of life (*g*=0.35, *n*=43), and general psychiatric distress (*g*=0.40, *n*=12) better than the control conditions including waitlist, assessment only, educational resources, placebo controls, and treatment as usual [24]. However, the smartphone interventions did not significantly differ from active interventions such as face-to-face or computerized treatment [24]. This study will provide a valuable addition to this growing body of research by exploring the feasibility and acceptability of behavioral health mobile applications among individuals with recent criminal justice contact.

This study directly responds to the above gaps in research through an extensive assessment of CSU utilization with stakeholders and the deployment of a mobile and technology-assisted aftercare intervention with individuals post-discharge from a CSU. Ultimately, this study will advance the understanding of CSU implementation, policies, and usage between law enforcement and CSU staff. This study is designed as a pilot study and results will be used to determine whether it is feasible to proceed with a large-scale RCT and how that RCT should be designed. Outcome criteria that will help determine whether to move forward with a larger trial include as follows: (1) are the results of the pilot study suggest positive effects on the treatment group? (2) does feedback from participants and stakeholders demonstrate both feasibility and acceptability of the intervention? (3) can a future RCT feasibly be implemented? and (4) are there potential future partners for a future RCT? The pilot study will assess the feasibility and acceptability of an aftercare intervention and explore the intervention’s ability to improve CSU patient outcomes in order to answer these critical progression criteria standards. The study is guided by three aims that collectively support the above goals. Aims 1 and 2 will be synthesized to inform Aim 3. These aims are as follows:

- Aim 1: Identify key elements and implementation factors for CSU dissemination and adoption.
- Aim 2: Develop an implementation guide and pilot test a CSU intervention with mobile and technology-assisted aftercare.
- Aim 3: Design a multi-site RCT study protocol. The potential study will be designed to compare the relative impact of CSUs with mobile and technology-assisted aftercare to CSUs without mobile and technology-assisted aftercare. Specifically, we will look at short and long-term outcomes of individual stabilization post-crisis, arrest rates, use of acute services, increased engagement with long-term treatment, and the cost-benefit of the intervention.

## Material and methods

### Aim 1 study design

#### Aim 1: Identify key elements and implementation factors for CSU dissemination and adoption

Aim 1 will use a qualitative case-study approach to investigate the feasibility and acceptability factors that may influence CSU dissemination and adoption. Study participants will include behavioral health professionals and law enforcement officers identified as CSU stakeholders due to their named direct or indirect involvement with CSU procedures, utilization, collaboration, or management. This sampling approach will allow for a representation of stakeholders that range from field staff up to leadership strengthening the breadth of our investigative lens. Interviews will be conducted by the research team. The research team members conducting interviews are master’s level social workers trained in interviewing skills, including how to talk with the research participant in an empathic manner that helps put them at ease, which is particularly important considering the personal nature of some of the research questions. Aim 1 interviews will focus on exploring the barriers and facilitators to CSU utilization and implementation from the perspective of law enforcement and mental health professionals and assess the decision making and procedural guidelines that impact CSU admission, collaboration, and success. Ultimately the data collected during Aim 1 will be analyzed to inform the development of an implementation guide for the mobile and technology-assisted program during Aim 2.

#### Sample

Purposive sampling will be used to recruit a specific sample of the intended population. Purposive sampling allows us to strategically identify and include law enforcement and mental health professionals who have experience with CSUs. The number of participants interviewed will be determined based on the relative size of each behavioral health and law enforcement agency. The total number of behavioral health agencies represented will be two. The number of law enforcement agencies represented will be seven. Based on the size of these nine agencies, and conversations about our project goals with their leadership personnel, we estimate approximately 80 interviews with CSU stakeholders but will interview up to 130 participants unless data saturation is reached after 80 interviews. Due to the unpredictable nature and demand of law enforcement and CSU-affiliated mental health staff schedules, our research team will implore several strategies to reach our sample goal. Strategies include recurring and flexible agency site visits, virtual interviewing options, and selection of optimal days for interviews based on leadership recommendations.

#### Recruitment

Stakeholders from behavioral health agencies will be recruited by establishing relationships with directors and supervisors of each agency and having them connect us to relevant department heads. Prospective sites will be met with and the research design, aims, and goals will be discussed and reviewed. Then, a memorandum of agreement will be signed to establish the researcher-practitioner collaboration. Recruitment dates will be coordinated over email and phone with department heads. During recruitment dates, we will travel to the agency and interview all available staff after reviewing confidentiality protections and a consent letter. Law enforcement stakeholders will be recruited by following the same site establishment methods as completed with the behavioral health stakeholders.

#### Measures

The research team developed the qualitative interviews to explore processes, barriers, and influences of CSU admission and service implementation. The interviews follow a semi-structured interview guide. The semi-structured interview guide for law enforcement and mental health stakeholders is shown in Table 1.

**Table 1:** Qualitative Questions for CSU Stakeholders

Semi-structured interview guide for CSU stakeholders	
Law Enforcement	Behavioral Health
1. What are you expected to do when you get a call to a scene with an individual in a mental health crisis? What are some possible outcomes to that call? 2. Please tell me about your experience with crisis stabilization units. 3. What does a referral to a CSU look like? a. Walk me through that process, starting with your interaction with the individual. 4. What are some things you consider before deciding to refer an individual to a CSU? a. What are some reasons you would not refer an individual to a CSU? 5. How did you learn about CSUs? a. Were you told about who is eligible for these programs at that time? 6. What could cause an individual to be denied services from a CSU? a. What happens after they are denied? 7. How often is your agency responding to mental health or substance use-related calls? 8. How would you describe the relationship between your department and the CSUs? 9. What would improve CSUs? 10. How does the utilization of CSUs impact the community? 11. How do CSUs impact public safety? 12. What conditions, if any, influence mental and substance-related calls in your area?	1. What does the process of crisis stabilization look like? a. What does the process look like once an officer brings someone in for a referral? 2. Who is eligible for services? a. How do you identify those individuals? b. How effective is the program at reaching qualifying individuals? c. What are some barriers to services? 3. What is the typical capacity of the CSU? a. How long do clients typically stay? 4. What types of services are provided at the CSU? 5. What type of community resources are used at the agency? 6. How does the community perceive the program? 7. What does success look like for the agency? a. How do you measure that? 8. What would change about the program? 9. How does the CSU discharge process look? 10. What are some, if any, of the characteristics observed in persons brought to the CSU? 11. Could a mobile aftercare program address any barriers to CSU success, if so, how would it help? 12. Does your agency have the means to deploy a mobile aftercare program to persons upon discharge from a CSU? 13. What, if any, mobile applications do your staff members recommend to clients with mental health and substance use related disorders? 14. How likely is your agency to incorporate mental and behavioral health mobile applications into the treatment plans with clients? What would this look like for your agency?

#### Data collection

Research team members will collect qualitative data by completing in-person qualitative interviews or focus groups with study participants. Whether a participant receives a qualitative interview or a focus group interview is determined by the individual’s preference and availability. These interviews will be audio-recorded, transcribed, de-identified, and the audio will be deleted upon completion. Qualitative interviews will be conducted one time with each CSU stakeholder. Interviews will occur in person at the representative agency the stakeholder is employed. If in-person is not an option, a HIPAA-protected zoom link may be utilized to conduct virtual interviews. Interviews will be conducted by the research team.

#### Analysis strategy

Data from Aim 1 will assist us in developing our implementation guide and pilot test in collaboration with our community partners. Qualitative data will be analyzed using descriptive statistics, meaning our research team will use frequencies, means, proportions/percentages to summarize data, and a qualitative content analytic approach. Consistent with Morgan’s [25] recommendations for qualitative content analyses and Hsieh and Shannon’s [26] directed qualitative content analytic approach, standard definitions of the concepts of interest are developed based on existing literature on feasibility and acceptability. For instance, barriers to feasibility may be organized into agency or role-specific concepts. As the analysis proceeds, additional codes may develop, resulting in a revision to the initial coding scheme [26]. The research team will begin by reviewing each interview line-by-line and identifying codes through a combination of inductive and deductive approaches. The research team will engage in frequent meetings to refine coding categories and ensure consistency. Initial coding will therefore indicate categories and themes, and as coding continues, the research team will continue to monitor and verify consensus between coders, and consistency and accuracy of codes. Once the research team has reached a consensus on each code, we will develop a codebook with the code definitions.

To ensure the study’s findings are credible, we will engage in several standard methods for establishing rigorous and trustworthy results. First, we will maintain an audit trail to document our decision-making process [27]. Additionally, we will use multiple coders. An initial assessment of intercoder reliability will be conducted using two independently coded interviews to establish an intercoder reliability score at a minimum of 0.60 or higher, as assessed by Cohen Kappa [28]. To ensure intercoder reliability is maintained, a random selection of 30% of the interviews will be independently coded to ensure that the kappa coefficient remains 0.60 or higher [28]. After each intercoder reliability assessment, coders will meet to discuss and resolve coding discrepancies. Examining patterns in the presence and absence of thematic categories will allow us to provide empirically grounded explanations for identifying key implementation factors for CSU adoption. Ultimately, data analysis from Aim 1 will be utilized during Aim 2 in the design and development of the mobile and technology-assisted aftercare implementation guide.

### Aim 2 study design

#### Aim 2: Develop an implementation guide of the CSU Mobile and Technology-Assisted Aftercare intervention and pilot test this implementation guide-based CSU intervention

For aim 2, we are developing an implementation guide to manualize the CSU mobile and technology-assisted aftercare intervention and then we will examine the impact of adding a mobile and technology-assisted aftercare intervention to persons diverted from jail and brought to a CSU by LEOs. The first objective of Aim 2 is to develop the implementation guide in order to manualize the intervention being tested and facilitate fidelity to the intervention across intervention deliverables. The implementation guide is informed by the qualitative data from Aim 1 interviews and ongoing interviews with CSU stakeholders throughout the implementation of the pilot intervention. Continuing to procure feedback from CSU stakeholders will allow the development of an implementation guide that is responsive to barriers faced by both law enforcement and mental health professionals.

The second objective of Aim 2 is to examine the trends in outcomes of individuals who are brought to a CSU by LEO decision and referral. Two study conditions will look at the trends in outcomes of individuals brought to CSUs. One study condition is an innovative mobile and technology-assisted aftercare intervention (treatment group), and the other condition is standard care at the existing CSUs (control group). Participants will be recruited into the study by a research team member and be randomly assigned to either the mobile and technology-assisted after intervention or to the standard care services.

Persons randomized into the standard care services group will receive the usual treatment services available to persons discharged from a CSU. These standard services may include a personalized discharge plan, a follow-up appointment within a month of discharge for evaluation, and connection to other agency services if applicable. A detailed description of standard services can be found in “Intervention description” section. Persons randomized into the mobile and technology-assisted aftercare group will receive standard services plus up to 26 sessions with a community support specialist (CSS) aftercare provider and six months of access to behavioral health mobile applications which are available on demand*.* The CSS is a mental health professional who travels to participants to provide mental health services including counseling, community referrals, motivational interviewing, solution-focused therapy, psychoeducation, and social support, up to once a week for 6 months. Participants randomized to this condition will also receive 24/7 access to evidence-based behavioral health mobile applications that are recommended based on the participant’s diagnostic status, learning style, and goals. The mobile applications are Moodtools-Depression Aid, Virtual Hope Box, Recovery Path, and Intellicare Hub. Each of these applications and the respective types of tools offered within are described in Table 2.

**Table 2:** Mobile applications

**Mobile application**	**Resources and tools offered**
Moodtools-depression aid	Psychoeducation Symptom tracking Customized safety plans Cbt activities Journaling activities
Intellicare hub	Psychoeducation Symptom tracking Goal setting Relaxation techniques Positive thinking activities
Virtual hope box	Coping skills Relaxation techniques Positive thinking activities
Recovery path	Psychoeducation Goal setting Coping skills Trigger management Referrals to recovery meetings Daily check-ins

#### Sample

The goal for Aim 2 is to recruit 24 adults; 12 individuals in the mobile and technology-assisted aftercare group and 12 individuals in the standard services group. These adults will be recruited equally across the three participating jurisdictions resulting in eight participants per county. Individuals brought to the CSU by law enforcement will be randomly assigned to one of two study conditions in equal proportions. To be eligible for participation in Aim 2, individuals must be age 18 or older, English speaking, will discharge to one of three participating counties and plan to remain in the county for 6 months, admitted to the CSU through law enforcement, consent, and the cognitive capacity to understand study participation and to consent. This sample size is strategically small due to testing the feasibility of accessing the needed sample size for future studies. With this pilot sample size, we will be looking at the feasibility of recruiting individuals in a vulnerable, highly transient, population, and retention trends. Both feasibility of recruitment and retention trends will impact our decision on a larger sample size in the subsequent RCT.

#### Recruitment

Research participants for Aim 2 will be recruited into the study at our partner mental health agency’s CSU prior to their discharge. CSU patients will be flagged as potential research participants by an agency staff member. This staff member flags CSU patients based on whether a patient meets the inclusion criteria. Once the research team is alerted of the potential participants, the research team will go on-site to ensure eligibility. Eligible CSU clients will be informed of the research study and provided details of participation by the research team member. Informed consent will be obtained after performing a cognitive assessment. The cognitive ability to consent will be determined through the brief capacity to consent screener, a tool that checks comprehension of participating in the study—cognitive capacity is re-assessed at subsequent data collection points when warranted. Potential participants will be given the option to consent to participate by the research team member at this time.

#### Randomization

In Aim 2, participants who will be recruited from the CSU will be randomized into either the control group (treatment as usual) or the intervention group (technology-assisted mobile aftercare) by the research team. Due to the short length of stay in a CSU, the research team will use a one-to-one randomization procedure and pre-randomize the pool of participants prior to recruitment. To do so, the research team will create an allocation table with the total participant slots (*n*=24) and then use a random sequence generator to pre-randomize each slot as either the control group or the intervention group. The randomized allocation table will be uploaded into our online database where the randomization status will only be visible to the research team member upon completing the consent process with a participant. Following these guidelines eliminates the potential for human error or bias into who receives the mobile aftercare intervention. Participants will be notified of their randomization status at the end of the consent and baseline data collection interview by the research team. It is necessary to inform the participants of their randomization group because the interventionist will contact participants in the treatment group within 24 hours to set up their first appointment.

#### Intervention description

The mobile and technology-assisted intervention is designed as an aftercare program for persons released from a CSU and addresses treatment retention barriers by combining in-person assistance and technology-aided tools. The aftercare program will be delivered to participants by Community Support Specialists (CSSs) who are employees of our partnering behavioral health agency and trained in the research study protocol. The aftercare program will be delivered up to once a week for 6 months or 26 sessions; the needs of each participant determine the frequency of sessions.

The mobile and technology-assisted aftercare program services will include the CSSs providing in-person assistance by meeting with participants at their homes or nearby public settings and providing participants with 24/7 access to behavioral health services through mobile applications. During sessions, the CSSs will provide various assistance, including social support, psychoeducation on behavioral health, and health management skills, and assist with the navigation of community resources, including housing, meeting basic needs, and other tangible supports that promote community stability. To provide support in-between session work, the CSSs will give guidance on using the mobile apps that are relevant to the participant. Additionally, CSSs will employ motivational interviewing and solution-focused brief therapy techniques in the sessions. The mobility of the aftercare program differs from standard services in which persons are expected to return to the behavioral health agency approximately 1 month after discharge from the CSU. The mobile and technology-assisted aftercare program is based on the Community Health Model (CHW) wherein mobile CHW solutions provide resources, counseling, education, and assistance to vulnerable and disadvantaged populations [29, 30]. Similar to CHW programs, a vital component of the CSS work is augmenting interventions at our partner mental health agency by providing community-based services and mobile app support to address client barriers to treatment directly.

The technology-assisted component of the mobile and technology-assisted aftercare program includes 24/7 access to behavioral health mobile applications. These apps are free for the participants and uploaded onto their own smartphones. If the participant does not have a reliable access to a smartphone or does not wish to have the apps uploaded to their phone, they will be provided a smartphone for free by the research team. Participants who are provided a smartphone will have the phone bill covered by the research team for the duration of their participation in the research study. Upon completion or termination of the study, the research team will cease paying the phone bill, but participants may keep the phone. The mobile applications serve to augment the in-person support provided by the CSS. The CSS will recommend one or more mobile applications from a menu of evidence-based applications based on the participant’s diagnosis and treatment goals. The intervention strictly differs from standard services in this aspect because standard services do not connect individuals to mental and behavioral health mobile applications.

Mobile applications incorporated into this intervention include Moodtools-Depression Aid, Virtual Hope Box, Recovery Path, and Intellicare Hub. All mobile-based applications have undergone thorough investigation in one or a combination of RCTs/clinical trials, were developed with mental health professionals, and have shown a positive impact on intended outcomes [31–36]. Participants will not be required to use the mobile applications. However, the participants will be encouraged to use the apps by the CSS. The CSS will incorporate dialogue and instruction on the utilization of the apps into the intervention sessions. Specifically, during sessions, the CSS will ask participants about their use of the apps, what they like and do not like, and provide information on how the app(s) may assist the participant in reaching their goals. The CSS will not record, track, or obtain any data from the mobile apps on the participant’s phones. If participants do not wish to use the apps, they will not have to. The research team will not track, record, or obtain any data from the apps on the participant’s phones. The research team will collect data on the use of the apps through an open-ended question measure, Qualitative Technology Use, described in “Measures” section. The above mobile applications were chosen for a variety of reasons, including their development standards, accessibility for download, and range of mental health and substance use-related tools and resources that are promising and apply to our intervention and population.

Standard care for persons discharging the CSU will include a personalized discharge plan and a follow-up appointment with the agency within a month for evaluation and continued mental health services. At the follow-up appointment, participants may be connected to other agency services. These services include outpatient counseling for behavioral and emotional problems, psychiatric services, group therapy, case management, rehabilitation services, homeless recovery services, employment services, medication-assisted treatment, group-based team-building activities, a primary care clinic, and forensic services. Individuals may also receive referrals for applicable healthcare services, access to residential and outpatient substance abuse treatment services, and access to a tablet.

#### Fidelity monitoring

Fidelity monitoring is a set of practices that help researchers measure how well an intervention is delivered and the extent to which it is delivered as intended. We will conduct fidelity monitoring to ensure participants receive the mobile and technology-assisted aftercare as it is designed and be confident that the CSSs have the necessary skills and knowledge to implement the services. Fidelity monitoring will be conducted with participants engaged with the mobile and technology-assisted aftercare intervention study condition. We adapted the fidelity monitoring tool used for this study from the Well-Being Development Program Fidelity Tool used in a multi-site randomized controlled trial of a behavioral health intervention designed for justice-involved individuals [37]. This adapted tool includes a measure that assesses motivational interviewing with the participants for sessions where this therapeutic technique is used. The research project manager will complete the on-site observation of the intervention. Due to the mobile nature of the intervention, the CSS will meet with the participants in various settings. The project manager will randomly select one session to monitor each week. After the session, the project manager will review the fidelity monitoring ratings with the CSS to give feedback. A CSS who scores lower on the fidelity ratings will receive additional training in the intervention to improve the likelihood that they can improve and maintain fidelity.

#### Measures

Measures for the Mobile and Technology-Assisted Aftercare Group and Standard Services Group include administrative records and direct data collection from participants. The primary outcomes for this feasibility study are participant recruitment, participant retention, feasibility, and the acceptability of the intervention. Participant recruitment will be measured by the number of eligible participants who consent to participate out of the total number of eligible participants. Participant retention will be measured by the proportion of the intervention completed by participants. Feasibility and accessibility will be assessed by triangulating the qualitative and quantitative data from study participants—the CSU professionals and patients.

 The secondary outcomes include individual stabilization and well-being, frequency of both acute and long-term treatment use, re-admission to a crisis stabilization unit, and arrest rates or re-encounters with law enforcement. The secondary outcomes will be assessed to find trends that will inform the future RCT. We will examine the feasibility of collecting the data, the sensitivity of the measures, estimates for effect size and sample size, and other factors that may impact the ability to detect outcomes. For the pilot study, the secondary outcomes are not meant to measure impact; instead, they are intended to examine trends in outcomes to inform the design of the intervention and study protocol for the future RCT. Secondary outcomes will be measured using screening questions, demographical information, clinical assessment tools, and baseline and follow-up interviews with each participant. Administrative data from the CSUs and law enforcement will be collected on client characteristics, including demographics (e.g., race, gender, age, county of residence), criminal history (e.g., current reason for law enforcement contact, prior arrests, and reasons for arrest, and prior CSU stays), and behavioral health status (e.g., diagnoses, prior and current treatment utilization, and medication-assisted treatment). Table 3 shows the measures related to each secondary outcome described below.

**Table 3:** Primary and secondary measures

**Primary outcomes**	**Measures**
Participant retention	Number of recruited participants who complete the intervention
Participant recruitment	Number of eligible participants who agree to participate out of the total number of eligible participants
Feasibility of intervention	Number of intervention sessions completed by each participant
Acceptability of intervention	Qualitative interviews with CSU stakeholders
**Secondary outcomes**	**Measures**
Individual stabilization and well-being	Brief Symptom Inventory Brief COPE Hearth Hope Index Trauma History Questionnaire Financial security Education and employment Education employment aspiration and satisfaction Network composition survey
Acute and long-term treatment use	Service assessment for children/adults California Psychotherapy Alliance Scale Qualitative CSU experience Qualitative technology use
Admission into CSU	Administrative data of re-admission into CSU
Arrest rates/re-encounters with law enforcement	Administrative data of arrest rates Lawbreaking Police-community interaction survey

The Brief Symptom Inventory [38] is a 53-item assessment covering nine dimensions through subscales: somatization, obsession-compulsion, interpersonal sensitivity, depression, anxiety, hostility, phobic anxiety, paranoid ideation, and psychoticism. Scores for each subscale range from 0 to 4, with higher scores meaning more symptoms reported. Reported internal consistency reliability ranged from *⍺*=0.75 to 0.89 [39], test-retest reliabilities ranged from 0.68 to 0.91, acceptable convergent validity, and construct validity [40].

The Brief COPE [41] assessment is a 28-item index that assesses coping self-efficacy and confidence coping with stress using an intensity scale of 1 to 4. The higher the score, the greater the coping self-efficacy. The Brief COPE has been found to have acceptable reliability *⍺*=0.81 [42].

The Herth Hope Index [43] is a 12-item measurement tool that assesses hope, self-efficacy, and the respondent’s outlook on life. The tool uses an intensity scale from 1 to 4, where the higher reported scores indicate a higher level of hope. The Herth Hope Index has been used in 10 countries and has shown acceptable reliability and acceptability across 13 studies [44].

The 25-item Trauma History Questionnaire (THQ) assesses the occurrence of specific traumatic experiences, including interpersonal violence, accidents and disasters, serious illness, traumatic loss, and criminal victimization. Participants will indicate if they have experienced the event with dichotomous “yes” or “no” questions. Respondents are then asked to self-report the age they were at the time of the incident and the frequency of the trauma. The THQ operationalizes the Diagnostic and Statistical Manual of Mental Disorders, 5th edition (DSM-5) criteria for trauma exposures. The THQ has been found to be a reliable measure of trauma exposure (3-month test-retest coefficients=.51–.91) and has strong convergent validity to other measures (*k*=.61–1.00) [45].

The eight-item financial security measure was conceptually created from the work of Mills et al. [46] and adapted for an RCT trial of a behavioral health-focused intervention for justice-involved individuals [47]. The measure assesses an individual’s ability to pay for one’s bills, basic needs, and potential unexpected expenses without difficulty. Various intensity scales (0 = none/never to 5 = all of the time) are included alongside dichotomous “yes” and “no” questions—the lower the score, the more financial stability.

The Education and Employment assessment is an 11-item questionnaire that measures past and current employment, sources of income, and education level. The Education Employment Aspiration and Satisfaction [48] survey is a 13-item assessment that measures an individual’s satisfaction with their current employment and education levels. The assessment uses an intensity scale of 1 to 4 for both the education and employment subscales. Higher scores for the education subscale indicate higher satisfaction and aspiration to obtain additional education. Higher scores on the employment scale indicate higher satisfaction and aspirations to improve their work situation. The scale has been shown to have acceptable reliability (*⍺*=0.79 to 0.90) across the four subscales and sufficient criterion-related validity [48].

The Network Composition Survey (NCS) was developed by the research team in response to a lack of validated social support surveys specific to populations with criminal justice system contact; NCS measures informational, emotional, and instrumental support and is in the process of being validated [(Curley E, Killian M, Renn T, Veeh C, Pettus C: Validation of a multidimensional social support measure for individuals who are incarcerated, Under review), 49]. NCS is a 23-item questionnaire that first has respondents identify three individuals in the participant’s “social network.” Then, the participant is asked an identical set of 23 questions about these three people to capture their perception of the presence of socially supportive characteristics. Various intensity scales are used (0= the least perception or never to 5 = the most perception of support or always). The higher the score, the greater the perception of social support. Dichotomous’ yes” and “no” questions are also used to capture participants’ perceptions of the social support characteristics within their social network.

The Service Assessment for Children/Adults [50] is a 45-item assessment tool that measures the use and need of services for cognitive, housing, relationships, health, education, job readiness, substance abuse, mental health, and life skills. The tool uses dichotomous “yes” and “no” questions, and then, respondents report how many times they have received the service. The measure was previously shown to have acceptable test-retest reliability, with kappa scores ranging from .75 to .94 [50].

The California Psychotherapy Alliance Scale [51] is a 24-item assessment that measures therapeutic alliance through goal consensus between the counselor and client, collaboration on counseling-related tasks, and emotional bonds. The measure utilizes an intensity scale of 1 to 7, where the higher the reported score, the greater the therapeutic alliance. The scale is reported to have internal consistency reliability of *⍺*=0.84 and sufficient criterion-related validity [52].

The research team developed the qualitative CSU questionnaire to explore the respondent’s experience while detained and receiving services within a CSU. The qualitative CSU experience questions are designed to assess the participant’s perceived helpfulness and impact from the CSU services which may affect the degree of stabilization respondents feel after discharge from the CSU. The Qualitative CSU experience is a five-item qualitative questionnaire. Qualitative CSU experience is a short survey containing both yes/no and open-ended qualitative questions. The questionnaire asks about the history of admission into CSUs, services received while at CSUs, and several questions regarding the participant’s opinion of their CSU stabilization experience. These opinion questions include what was helpful during their stay, what was not beneficial, and what they would like to change about the program.

The research team developed the Qualitative Technology Use questionnaire to capture the respondents’ opinion on and use of the behavioral health mobile applications. The Qualitative Technology Use is a short four-item survey and contains qualitative and quantitative questions. The questions are designed to address the feasibility and acceptability of the incorporation of mobile applications into our intervention. To do so, questions assess the frequency of use and what participants like and do not like about each app they use. Both treatment and control participants will be asked if they currently use mobile applications to assist with mental, behavioral, or substance use symptoms/treatment. Treatment group participants will be asked if they use any of the recommended apps or any other mobile apps they may use for behavioral health, mental health, and substance use assistance.

Our partnering mental health agency will collect administrative data to record any points of contact or engagement with participants. Administrative data will include re-admissions into CSUs, CSU admission and discharge criteria, number of contact attempts by the mental health agency, type and number of services offered to the participant, and engagement in and retention in acute and long-term treatment by the participant.

The Lawbreaking assessment is a 10-item questionnaire that is a dichotomous “yes” or “no” survey in which participants are asked about lawbreaking behavior. The Lawbreaking assessment was created by the research team and is based on two self-reported crime surveys [53, 54]. The measure asks if they had contact with police, if they participated in an activity that would have resulted in legal consequences had they been caught, or if they failed to pay child support.

The Police-Community Interaction Survey [55] is a 30-item survey that captures reported interactions between respondents and law enforcement. The survey includes dichotomous “yes” or “no” questions where respondents identify if the police officer did a specific action and intensity scales ranging from 1 to 4, where higher reports indicate more satisfaction with the police interaction. The measure has good reliability with subscales above >0.70 and construct validity [55].

#### Data collection

Data collection with recruited participants in the treatment and control groups will occur approximately four times throughout participation. The baseline interview will be conducted immediately following enrollment and consent before the participant discharges from the CSU. The remaining three data collection points include the complete data collection toolset. The first data collection sessions (T1) will occur 2 weeks post-discharge from the CSU. The second data collection point (T2) will be 3 months post-discharge from the CSU. Lastly, the third data collection session with recruited participants will occur 6 months post-discharge from the CSU (T3). These data collection sessions will be conducted at the participant’s home or a nearby public location that the participant requests.

Data from recruited participants will be collected using validated assessment tools, qualitative surveys, and administrative data. These data collection methods will be consistent throughout T1–T3 sessions for the mobile and technology-assisted group and the standard services group. However, the qualitative CSU experience survey may vary based on the participant’s situation. Specifically, the CSU experience survey will be collected only at the T1 interview unless a participant is readmitted to the CSU. If a participant is readmitted to the CSU, the CSU experience survey will be re-asked at the data collection interview following the CSU admission.

Administrative data will be collected for 6 months following enrollment. Additional administrative data will be collected from law enforcement agencies to identify arrests and police contact of participants. Although we will not be assessing for causality in the current proposal, it is critical for us to pilot the collection of these data as they are integral to the follow-up multi-site RCT.

#### Analysis strategy

Data collected from the Mobile and Technology-Assisted Aftercare and Standard Services Groups will allow researchers to gather preliminary data comparing those that receive standard CSU services to those that receive CSU services plus the mobile and technology-assisted aftercare support. Patterns will be monitored to assess the primary and secondary outcomes listed in Table 3. A mixed-data analysis plan, including descriptive statistics (means, proportions, frequencies, and percentages) and bivariate analyses, will be used to capture the range of qualitative and quantitative data present in this study’s primary and secondary outcomes. The study is not designed to detect differences in clinical outcomes; thus, the focus of the analysis provides preliminary information on trends in outcomes and some potential underlying contributors to those trends. These data will provide essential information for advancing knowledge of CSUs and information necessary to inform the future RCT. Qualitative data related to the primary outcomes will be analyzed using the approach described in the Aim 1 analysis strategy. Quantitative analysis for primary outcomes will be conducted using a comparative analysis both within groups and across time. The bivariate analysis will be used to assess correlations and regressions. Additionally, preliminary ANOVAs will be used to assess the trends of participants. Qualitative and quantitative data will be combined using a sequential mixed methods design. Equal weight will be given to the qualitative and quantitative data sources. Data will be reviewed and analyzed for any significant variations that can help assess trends in participants over time and across groups through mean comparison tests.

Mixed methods analysis will be used to assess both primary and secondary outcomes. We will integrate the findings from each of the quantitative and qualitative components [56] by conducting comparative analyses both within and across groups, over time, so that we may examine differences at the county level in terms of quality and fidelity of the intervention. Data will be integrated with quantitative outcome measures, fidelity data, and qualitative interviews using a sequential mixed methods design with equal weight given to qualitative and quantitative data sources [56, 57]. Data will be assessed for significant variations, and qualitative data is used to help us understand quantitative data trends when warranted.

### Aim 3 study design

#### Aim 3: Design a multi-site RCT to compare the relative impact of CSUs with mobile and technology-assisted aftercare to CSUs without mobile and technology-assisted aftercare

The goal of Aim 3 is to design a large-scale multi-site RCT to compare CSUs with and without mobile technology-assisted aftercare on short and long-term outcomes. It is anticipated that the outcomes in this future RCT will include individual stabilization post-crisis, arrest rates, use of acute services, and increased engagement with long-term treatment

To reach Aim 3, the qualitative data from CSU stakeholders in Aim 1, and data from the pilot trial of the CSU aftercare intervention in Aim 2, will be synthesized to inform and develop a study protocol for the future large-scale RCT. Following the guidelines from the CONSORT extension to pilot trials, data from Aims 1 and 2 will be used to inform the feasibility and suggest critical areas where further development or modification may be required [58]. For instance, data from Aim 1 will explore barriers and facilitators to intervention implementation for agencies and the perceived acceptability of the intervention for our target population and stakeholder procedures. Analysis of Aim 2 data will inform sample size details, referral, appropriate randomization procedures, retention and attrition challenges, and whether randomizing to follow-up mobile supports is the correct variable to randomize on or if there is another more appropriate randomizing variable. Prespecified progression criteria will be assessed from our pilot study to determine whether to proceed with the development of the future RCT. Our progression criteria include the following: (1) do the results of the pilot study suggest positive effects on the treatment group?; (2) did feedback from participants and stakeholders demonstrate the feasibility and acceptability of the intervention?; (3) can a future RCT feasibly be implemented?; and (4) are there potential future partners for a future RCT?

Recommendations to avoid common mistakes during small-scale pilot trials like this one include capturing data on acceptability and feasibility and information on secondary outcomes [59]. Our data on participant outcome trends directly responds to this recommendation. Outcome trends observed in participants will include the use of acute services, engagement with long-term treatment, arrest rates, and multiple factors of individual stabilization (i.e., financial stability, employment stability, perceived social support, and psychological well-being). Ultimately, this data will provide a greater understanding of our target population, including common challenges and experiences with the intervention. This process enables careful refining of the intervention for Aim 3 before a large-scale RCT.

## Study timeline

This study will take approximately 2 years to complete. The estimated time necessary to complete each of the three aims is detailed in Table 4. This study does not operate on a strict linear timeline, and the data collection and analysis of Aims 1 and 2 may overlap or occur coincidingly. However, Aim 3 will not begin until Aims 1 and 2 have been completed.

**Table 4:** Study timeline

**Study timeline**	2 mths	4	6	8	10	12	14	16	18	20	22	24
**Aim 1**												
Research preparation and stakeholder establishment	x											
Qualitative interviews w/ CSU stakeholders and iterative analysis		x	x	x	x	x	x	x	x	x		
**Aim 2**												
Pilot test activities				x	x	x	x	x				
Data analysis and implementation guide								x	x	x		
**Aim 3**												
Study protocol development and completion										x	x	x

## Discussion

Because short-term crisis centers, like CSUs, are increasingly being implemented in communities, it is critical to understand how these facilities operate as a jail alternative for law enforcement who have frequent contact with persons experiencing a behavioral health crisis [11, 12]. Similarly, research must discern CSU processes and how community care systems may minimize subsequent contacts with law enforcement and re-admission into CSUs for CSU patients. This study responds to this need by initiating a feasibility study examining critical CSU implementation and adoption elements while piloting an aftercare program for persons brought to the CSU by choice of law enforcement.

Conducting and exploring the feasibility and acceptability of CSU aftercare services is a critical first step in identifying how to implement CSUs as alternatives to arrest realistically and most effectively in diverse jurisdictions across the country. This study enhances our feasibility and acceptability data by engaging perspectives from a variety of stakeholders involved in CSU processes, including law enforcement, behavioral health staff, and CSU patients. Feasibility and acceptability data will come from a combination of qualitative interviews and quantitative analysis of the deployment of intervention-specific techniques and outcomes trends.

This study comes at a crucial moment in time as policymakers seek police reforms and alternative responses to issues that are more closely related to public health rather than public safety. Results from this study will advance the identification and dissemination of evidence-driven CSUs and yield three important products: (1) feasibility evaluation results that address jurisdictional variation and establish important feasibility guidance for practitioners and policymakers, (2) an implementation guide for future refinement and adopters of CSUs initially to be used to pilot test the CSU with follow-up mobile and technology-assisted aftercare, and (3) study protocol design for a future large-scale RCT of CSUs with follow-up mobile and technology-assisted aftercare. If mobile and technology-assisted aftercare pilot intervention demonstrates significant feasibility and acceptability, we are prepared to develop a large-scale RCT. An expansive RCT will further the evaluation of CSUs and enable a more generalizable analysis of CSU aftercare services on the persons who are a recipient of them. Understanding the effects of CSU aftercare services on persons may guide policy and national understanding of effective criminal justice diversion for persons with behavioral health disorders.

## Data Availability

The datasets used and/or analyzed during the current study are available from the corresponding author on reasonable request*.*

## Abbreviations

CSU: Crisis stabilization unit
CSS: Community support specialist
LEO: Law enforcement officer
CHW: Community health worker
RCT: Randomized controlled trial
